# Peroxisome proliferator-activated receptors (PPARs) are potential drug targets for cancer therapy

**DOI:** 10.18632/oncotarget.19610

**Published:** 2017-07-27

**Authors:** Qian Gou, Xin Gong, Jianhua Jin, Juanjuan Shi, Yongzhong Hou

**Affiliations:** ^1^ Department of Oncology, Affiliated Wujin People's Hospital, Jiangsu University, Changzhou, 212017, PR China; ^2^ Institute of Life Sciences, Jiangsu University, Zhenjiang, 212013, PR China

**Keywords:** PPARs, ligands, cancer therapy, cell signaling

## Abstract

Peroxisome-proliferator-activated receptors (PPARs) are nuclear hormone receptors including PPARα, PPARδ and PPARγ, which play an important role in regulating cancer cell proliferation, survival, apoptosis, and tumor growth. Activation of PPARs by endogenous or synthetic compounds regulates tumor progression in various tissues. Although each PPAR isotype suppresses or promotes tumor development depending on the specific tissues or ligands, the mechanism is still unclear. In this review, we summarized the regulative mechanism of PPARs on cancer progression.

## INTRODUCTION

As the nuclear hormone receptor, peroxisome-proliferator-activated receptors (PPARs) consist of PPARα, PPARδ and PPARγ, which are ligand-activated transcription factors. Ligand binding and activation of PPARs heterodimerize with retinoid X receptor (RXRs) and regulate gene transcription. Although PPARs/RXRs bind to the peroxisome-proliferator response element (PPRE, consensus sequence 5′-AGGTCA N AGGTCA-3′, N being any nucleotide) of target gene promoter regions, the each PPAR isotype consensus PPRE motif is different [1–5]. PPARs play a critical role in regulation of obesity, diabetes, atherosclerosis and cancer [6–9]. Even though the PPARs family contains PPARα, PPARγ and PPARδ, they serve as different functions in tumor development. Increasing evidences show that PPARα [2, 10–12] or PPARγ [7, 8, 13] inhibits tumor progression, which acts as tumor suppressors, while some reports show that PPARα is associated with tumor progression [14–16]. In contrast, PPARδ promotes tumor development [3, 6, 17]. PPARδ is associated with ulcerative colitis (UC) and Crohn's disease (CD), which is involved in the progression of colorectal cancer (CRC) [18, 19]. Endogenous or synthetic ligands can activate PPARδ resulting in inflammation and cancer depending on the specific ligands and tissue types [20–22]. Therefore, PPARs can be activated by endogenous or synthetic ligands, subsequently PPARs dependently or independently regulate tumor progression depending on the conditions. In this review, we discussed the progress of PPARs on tumor development.

### PPARα

**Figure 1:**
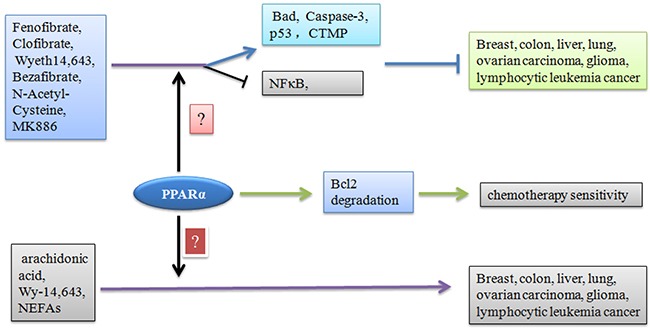
Effect of PPARα ligands on tumor progression Agonists regulate different types of tumor progression in a PPARα dependent or independent manner. In addition, PPARα destructs Bcl2 function leading to increased chemotherapy sensitivity of cancer cells.

Lack of PPARα expressions are associated with shorter breast cancer-specific survival [23]. Our previous investigation shows that PPARα induces Bcl2 degradation leading to increased SW480 colonic cancer cell apoptosis in response to chemotherapeutic agents [10]. Glut1 plays a critical role in glucose uptake to regulate cancer cell metabolism, which is widely expressed in most types of cancer cells [24, 25]. PPARα can directly inhibit Glut1 transcription by binding Glut1 potential PPRE motif [2]. The synthetic ligands of PPARα including fenofibrate, clofibrate and wyeth14,643 suppress cell proliferation by inducing apoptosis and cell cycle arrest involved in inhibition of NFκB [26] and activation of caspase-3 [26, 27]. More importantly, the combination of wyeth-14,643 and bezafibrate significantly suppresses lung cancer cell growth [12]. In addition, N-Acetyl-Cysteine (NAC)/PPARα signaling suppresses Non-small cell lung cancer (NSCLC) cell growth involved in increased the expression of p53 [28]. Although fenofibrate promotes breast cancer cell apoptosis via NFκB-mediated activation of caspase-3 and expression of Bad, which is independent of PPARα activity [27], clofibrate or wyeth14,643 induces hepatocarcinoma HepG2 cell apoptosis [29] and inhibits tumor progression [11] in a PPARα-dependent manner. Moreover, fenofibrate suppresses Huh7 hepatocarcinoma cell proliferation by increasing C-terminal modulator protein (CTMP) expression [27]. In addition to the inhibition of PPARα on tumor progression, PPARα^−/−^ mice inhibit tumorigenesis involved in increased endogenous angiogenesis inhibitor thrombospondin-1(TSP-1) [14]. Endogenous PPARα ligand arachidonic acid (AA) enhances breast cancer cell proliferation by up-regulation of cyclin E levels [30]. Nesterified fatty acids (NEFAs) activate PPARα-mediated hepatocarcinogenesis [31]. Therefore, PPARα antagonist MK886 and NXT629 inhibit chronic lymphocytic leukemia (CLL) cell proliferation [15, 16]. Other reports show that clofibrate promotes ovarian and prostate cancer progression independent of PPARα [32]. These findings suggest that different agonists play diversity functions on tumor progression, sometimes they serves as reverse roles, which depends on the tissue types or PPARα ligands (Figure 1). The discrepancy is associated with the dose of ligands or types of these ligands. Therefore, it is necessary to synthesize the suitable ligands for cancer treatment, which will provide a new drug target for cancer treatment.

### PPARδ

**Figure 2:**
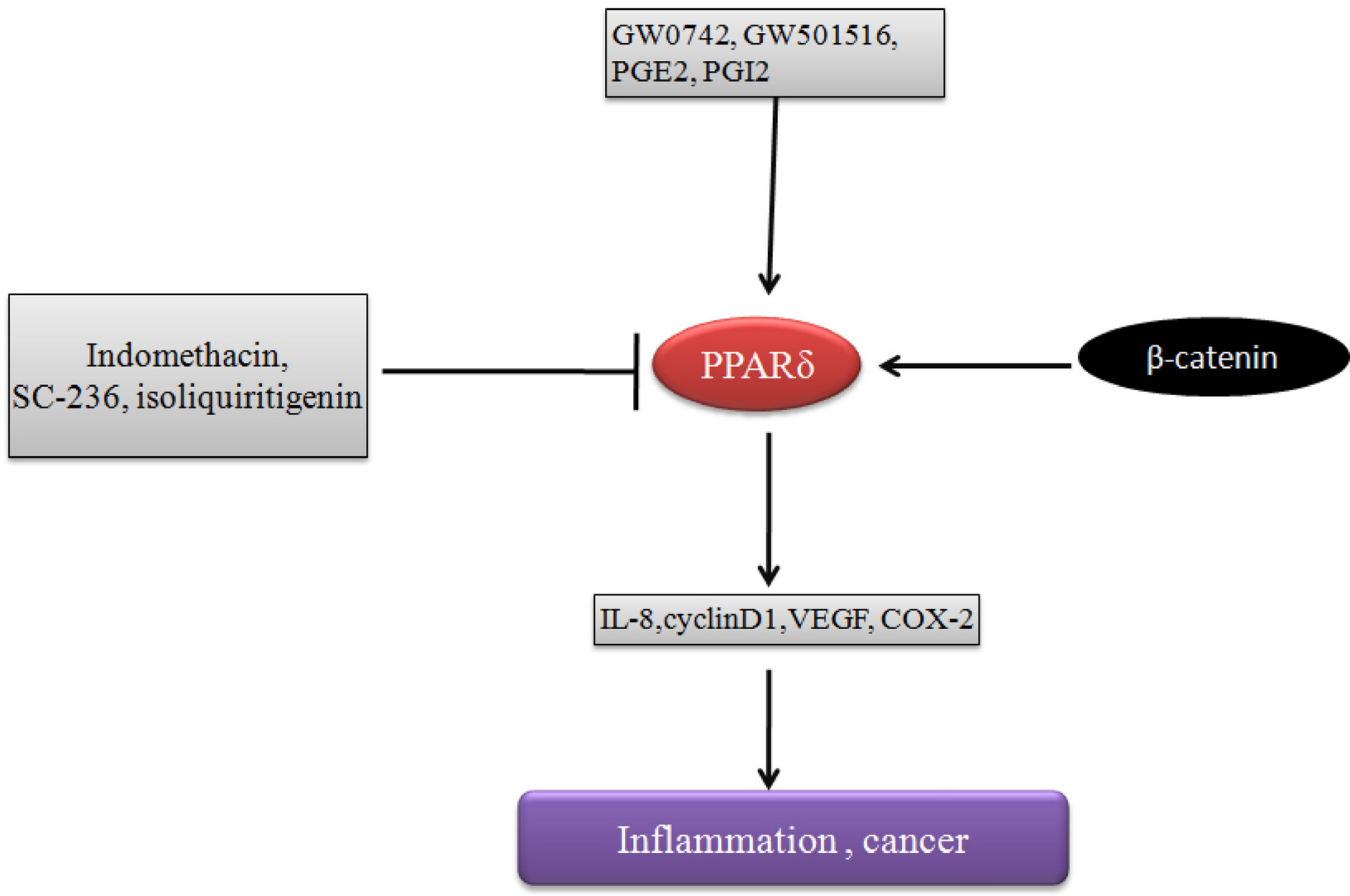
PPARδ promotes tumor development Agonists of PPARδ promote inflammation and tumor development by inducing cyclin D1, IL-8, VEGF, COX-2 expression, which is inhibited by the inhibitors of COX-2 such as indomethacin, SC-236, isoliquiritigenin.

Increasing literatures show that aberrant expression of PPARδ is associated with pro-inflammatory response and tumor progression [3, 17]. Consistent with this, overexpression of PPARδ causes AOM-induced colon tumorigenesis [33], and ultraviolet (UV)-induced PPARδ expression leads to Src activation and EGFR/ERK signaling-mediated skin cancer in mice. In contrast, PPARδ^−/−^ mice inhibit DSS-induced colonic inflammation and colitis-associated tumor growth [20], which is associated with inhibition of VEGF expression [34]. Since 14-3-3ε interacts with Bad leading to inhibition of cell apoptosis [35], PPARδ activation by PGI2, COX-2-derived prostacyclin, directly induces 14-3-3ε gene expression [36]. COX-2 inhibitors (COXIBs, indomethacin, SC-236 and isoliquiritigenin) suppress PPARδ signaling-mediated cell proliferation and tumorigenesis [17]. Wnt/β-catenin/signaling promotes tumorigenesis by inducing PPARδ expression [18, 37], which is associated with PPARδ-mediated cyclin E1 and VEGF expression [38–40]. In contrast, APC inhibits PPARδ transcription activity [18, 41]. PPARδ induces VEGF expression leading to PPARδ activation by VEGF/PI3K/Akt pathway [40, 42, 43], suggesting that activation of PPARδ undergoes a feedback loop [20, 40]. In contrast, PPARδ-mediated tumor development is inhibited by nitric oxide donating aspirin (NO-ASA) [44]. In addition to PPARδ-mediated tumor progression, PPARδ ligand GW0742 reduces colon or breast cancer event [45, 46], this event is reversed in PPARδ^−/−^ mice [47]. PPARδ promotes HARS-induced senescence leading to inhibition of tumorigenesis [48]. Consistent with this, silence of PPARδ results in cell proliferation and tumor growth [49]. Clinical observations show that although PPARδ protein levels are lower in human colon adenocarcinomas [50], high PPARδ protein levels are benefit of colorectal cancer patients [51]. However, increasing evidences show that PPARδ promotes tumor growth [17, 20, 21, 34, 39, 40]. Taken together, PPARδ regulates tumor progression involved in multiple signaling pathways (Figure 2). It needs to further determine the physical mechanism of PPARδ on tumor development.

### PPARγ

**Figure 3:**
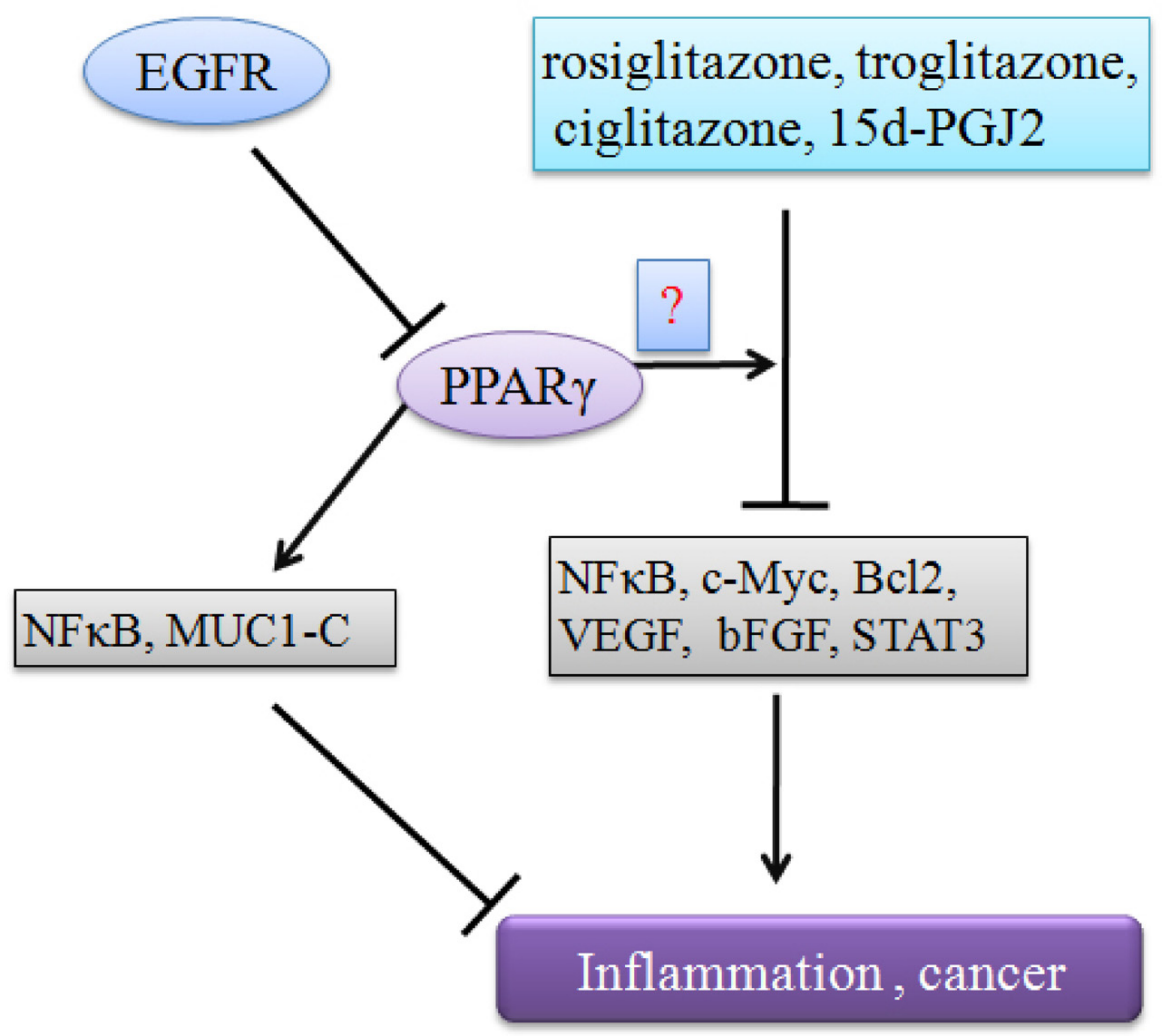
PPARγ negatively regulates tumor progression Agonists regulate tumor progression in a PPARγ dependent or independent manner, which are involved in inhibition of NFκB, c-Myc, Bcl2, VEGF, bFGF, STAT3. In addition, EGFR can terminate PPARγ antitumor function.

PPARγ plays an important role in inflammation, glucose metabolism and cancer [7–9]. While some clinical observations show that PPARγ expression levels are high in advanced prostate cancer (APC) tissues, ovarian, prostate and testicular carcinoma tissues [52–55], it is unclear whether the high levels of PPARγ correlate with favorite outcome in cancer patients. However, other clinical observations show that high PPARγ protein levels are benefit of colonic cancer, cervical carcinoma, follicular thyroid tumor, and esophageal cancer [9]. Consistent with this, overexpression of PPARγ inhibits cell proliferation and tumor growth, but this is reversed in PPARγ silenced cancer cells or activated EGFR signaling [7–9, 13]. PPARγ natural ligand 15-Deoxy-Δ-Prostaglandin J2(15d-PGJ_2_) induces cell apoptosis involved in inhibition of NFκB (nuclear factor-κB) [56]. In addition, some synthetic ligands such asrosiglitazone, troglitazone and ciglitazone suppress cell proliferation by inducing apoptosis, that is involved in reduced c-Myc, Bcl2, VEGF, and bFGF expression [9]. Moreover, ciglitazone increases the effective of cisplatin on human ovarian cancer treatment [57]. However, ciglitazone and troglitazone suppress ovarian cancer cell proliferation as well as rosiglitazone induces MCF-7 breast cancer cell or pancreatic cancer cell apoptosis independent of PPARγ activity [58–60]. In addition, 15d-PGJ2 and rosiglitazone independent of PPARγ inhibit Janus Kinase (JAK)- signal transducer and activator of transcription (STAT) pathway [61]. These findings suggest that although some ligands show anti-tumor activity, they are independent of PPARγ activity with different mechanism (Figure 3). In addition, overexpression or silence of PPARγ suggests that it indeed inhibits tumor growth [7–9]. Therefore, there is a need to develop and test selective PAPRγ ligands.

### Potential therapeutic targets for cancer

Increasing literatures show that PPARα or PPARγ can inhibit tumor progression by multiple pathways, which can be the potential therapeutic targets for cancer treatment, while some agonists suppress tumor progression in a PPARα/γ- independent manner (Figure 1, Figure 3). In contrast, PPARδ can promote tumor progression, so the antagonists of PPARδ may be the potential therapeutic targets for cancer treatment (Figure 2). Taken together, there is a need to develop and test selective PPARs ligands because of some agonists or antagonists independent of PPARs activity on effect of tumor development.
